# Integrative miRNA and mRNA analysis in penile carcinomas reveals markers and pathways with potential clinical impact

**DOI:** 10.18632/oncotarget.14783

**Published:** 2017-01-21

**Authors:** Hellen Kuasne, Mateus C. Barros-Filho, Ariane Busso-Lopes, Fábio A. Marchi, Maisa Pinheiro, Juan J. M. Muñoz, Cristovam Scapulatempo-Neto, Eliney F. Faria, Gustavo C. Guimarães, Ademar Lopes, José C. S. Trindade-Filho, Maria Aparecida C. Domingues, Sandra A. Drigo, Silvia R. Rogatto

**Affiliations:** ^1^ CIPE-A. C. Camargo Cancer Center, São Paulo, Brazil; ^2^ Department of Urology, Faculty of Medicine, São Paulo State University-UNESP, Botucatu, São Paulo, Brazil; ^3^ Department of Pathology, Americas Centro Oncológico Integrado, Rio de Janeiro, Brazil; ^4^ Department of Urology, Barretos Cancer Hospital, Barretos, São Paulo, Brazil; ^5^ Department of Urology, A. C. Camargo Cancer Center, São Paulo, Brazil; ^6^ Department of Pathology, Faculty of Medicine, São Paulo State University-UNESP, Botucatu, São Paulo, Brazil; ^7^ Department of Clinical Genetics, Vejle Sygehus, Vejle, Denmark; ^8^ Institute of Regional Health, University of Southern Denmark, Denmark

**Keywords:** penile carcinoma, micro-RNA, integrative analysis, HPV infection, lymph node metastasis

## Abstract

Penile carcinoma (PeCa) is an important public health issue in poor and developing countries, and has only recently been explored in terms of genetic and epigenetic studies. Integrative data analysis is a powerful method for the identification of molecular drivers involved in cancer development and progression. miRNA and mRNA expression profiles followed by integrative analysis were investigated in 23 PeCa and 12 non-neoplastic penile tissues (NPT). Expression levels of eight miRNAs and 10 mRNAs were evaluated in the same set of samples used for microarray and in a validation set of cases (PeCa = 36; NPT = 27). Eighty-one miRNAs and 2,697 mRNAs were identified as differentially expressed in PeCa. Integrative data analysis revealed 255 mRNAs potentially regulated by 68 miRNAs. Using RT-qPCR, eight miRNAs and nine transcripts were confirmed as altered in PeCa. We identified that *MMP1, MMP12 and PPARG and hsa-miR-31-5p, hsa-miR-224-5p, and hsa-miR-223-3p* were able to distinguish tumors from NPT with high sensitivity and specificity. Higher MMP1 expression was detected as a better predictor of lymph node metastasis than the clinical-pathological data. In addition, *PPARG* and *EGFR* were highlighted as potential pathways for targeted therapy in PeCa. The analysis based on HPV positivity (7 of 23 cases) revealed five miRNA and 13 mRNA differentially expressed. Although in a limited number of cases, HPV positive PeCa presented less aggressive phenotype in comparison with negative cases. Overall, an integrative analysis using mRNA and miRNA profiles revealed markers related with tumor development and progression. Furthermore, *MMP1* expression level was a predictive marker for lymph node metastasis in patients with PeCa.

## INTRODUCTION

Penile carcinoma (PeCa) is an aggressive and mutilating disease with high incidence in developing countries, with few therapeutic options available and high morbidity [1–3]. Several risk factors have been identified in PeCa etiology, including poor penile hygiene, phimosis and human papillomavirus (HPV) infection [4, 5]. A recent analysis of cancer registries showed that PeCa patient survival has not improved in Europe nor United States in the last 20 years [6].

The surgical approaches used for the management of PeCa patients are total or partial penectomy, which are associated with high morbidity [7]. The main prognostic factor in PeCa is the presence of lymph node metastasis, which is reported in 20 to 65% of cases [8, 9]. The overall five-year survival in patients with lymph node metastasis is 27% [10]. Novel molecular markers in association with lymph node metastasis [11], disease free survival or overall survival [12–14], recurrence and perineural invasion [15] have been described in PeCa. However, to date, none of them are used in the clinical practice to predict lymph node metastasis and prognosis.

Messenger RNA (mRNA) and micro-RNA (miRNA) expression signatures have diagnostic, prognostic and predictive values in a number of diseases [16]. To our knowledge, two reports described mRNA profiles in PeCa. In 56 PeCa samples, Kroon *et al*. [17] reported a 44-probe classifier that predicted lymph node metastasis. However, this classifier was not able to predict lymph node metastasis in an independent set of cases. In a previous study by our group, integration of the transcriptome of 33 PeCa samples with DNA methylation profiles [18] revealed several dysregulated oncogenic pathways associated with PeCa development and progression.

To date, five reports have described the differential expression of miRNAs in PeCa. Our group has previously reported that down-expression of *SLC8A1*, mediated by *hsa-miR-223*, promotes lower intracellular calcium concentrations, reduced apoptosis and increased cell proliferation in penile tumors [19]. Recently, *hsa-miR-218* and *miRNA-146a* down-expression and EGFR overexpression were reported as associated with high-risk HPV penile tumors [20, 21]. Using next generation sequencing, Zhang *et al*. [22] identified 56 differentially expressed miRNAs when comparing 10 matched PeCa with adjacent non-cancerous tissues. Hartz *et al*. [11] reported a miRNA-based signature associated with unfavorable prognosis in 24 PeCa and concomitant inguinal lymph node metastasis. Although these studies have contributed to our understanding of the disease, none have described an integrated analysis using miRNA and mRNA data in matched samples.

Approaches that integrate multiple *omic* profiles (i.e. mRNA and miRNA) provide a meaningful and comprehensive understanding of the biological processes involved in cancer development and progression. In addition, they may identify the genes that drive tumorigenesis, which have the potential to be applied in translational oncology [23–25].

In this study, integrated miRNA and mRNA profiles from the same set of PeCa samples were investigated, in order to gain insight into the mechanisms of penile carcinogenesis. In addition, miRNA and mRNA data were evaluated according to clinical and pathological characteristics, including lymph node metastasis and HPV infection status.

## RESULTS

### Distinct miRNA and mRNA expression profiles in PeCa

The unsupervised clustering analysis using miRNA expression data revealed two clusters separating tumors (*N* = 23) from non-neoplastic penile tissues (NPT = 12) (Supplementary Figure 1). The miRNA profile was composed by 81 differentially expressed miRNAs in PeCa (17 down-expressed and 64 overexpressed, Supplementary Table 1) (*P-value* < 0.01 and FDR < 5%). *hsa-miR-31-5p* showed the highest expression levels (FC = 352.4) in tumors compared to normal tissues, while *hsa-miR-891a-5p* presented the lowest expression levels (FC = –149.3).

The mRNA expression analysis comparing 23 PeCa samples with NPT revealed 2,697 differentially expressed transcripts (947 overexpressed and 1,750 down-expressed, CI = 99.9%, fold-change > 2).

**Figure 1:**
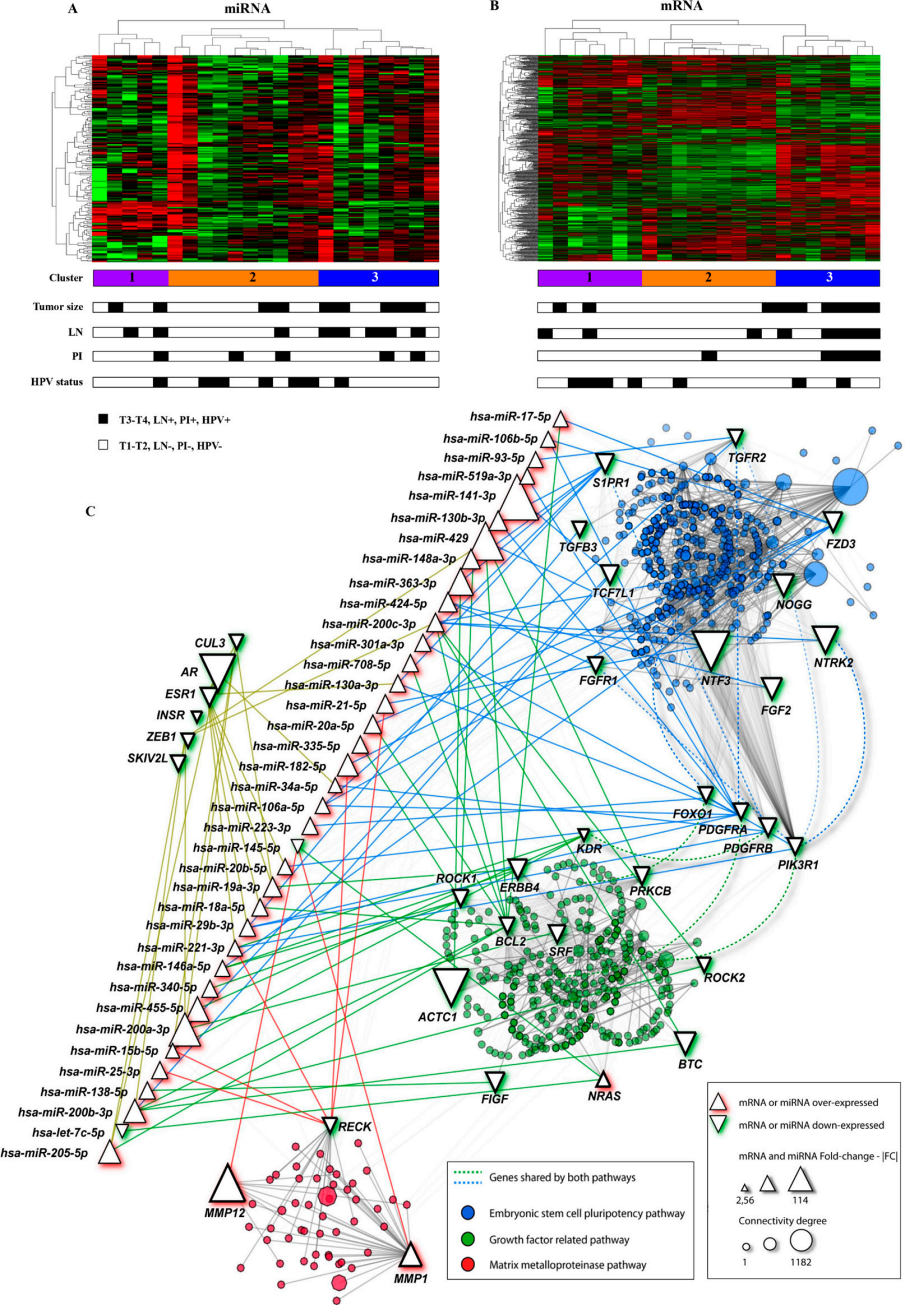
mRNA and miRNA unsupervised hierarchical clustering and pathway analysis with the genes found in the integrative analysis (**A**) The miRNA cluster 3 (blue color) was enriched with cases with lymph node metastasis (LN) and T3-T4 penile tumors. (**B**) mRNA cluster 3 (blue color) was mainly composed by cases with poor prognosis features: larger tumors (T3 and T4), LN and perineural invasion (PI). Most of the samples were HPV negative (miRNA and mRNA cluster 3 presented one and two HPV positive samples, respectively). (**C**) miRNA and mRNAs interaction networks in penile carcinomas (NAViGaTOR version 2.3). Circles in blue, red and green represent the mRNA belonging to the main canonical pathway altered in PeCa. Edges indicate the interaction between genes and miRNA according to the pathways (blue, red and green). Interactions among genes from different pathways are represented by non-continue edges. Triangles in the top left indicate the genes with highest number of interactions in PeCa. *AR* presented the highest number of interactions with other genes (1,182). Growth factor related pathway highlighted genes belonging to EGFR, VEGF and PDGF pathways. PI: Perineural invasion; LN-: absence of lymph node metastasis; LN+: lymph node metastasis confirmed by pathological analysis. T1 to T4: Tumor size.

Potential molecular signatures related with prognosis in PeCa were investigated for tumor samples using unsupervised hierarchical clustering analyses for both, miRNA and mRNA profiles. Three main clusters were detected for the PeCa samples according to miRNA profiles (Figure 1A). Cluster 3 (eight cases) was mainly enriched by the tumors with aggressive features (five cases with T3-T4 tumor size and five with lymph node metastasis). A similar analysis was performed with mRNA profiles (Figure 1B), which also revealed three clusters, where cluster 3 (seven cases) was comprised of patients with poor prognosis (six cases with T3-T4 tumor size, five with lymph node metastasis, and four with perineural invasion). Although not statistically significant, cluster 3 for both miRNA (*P* = 0.39) and mRNA (*P* = 0.49) profiles were enriched with patients with shorter overall survival (Supplementary Figure 2).

### Integration of miRNA and mRNA expression profiles reveals potential disrupted pathways in PeCa

Integrative analysis was performed using 81 differentially expressed miRNAs and 2,697 mRNAs. Based on predicted (mirWalk 2.0) and/or experimentally validated interactions (miRTarBase), 68 miRNAs that potentially regulate 255 mRNAs were identified (Supplementary Table 2), representing 598 miRNA/mRNA interactions with negative correlation (r Spearman < 0) and inverted fold change (Supplementary Figure 3).

The main canonical pathways detected by IPA and confirmed by KOBAS 2.0 revealed enrichment of the Human Embryonic Stem Cell Pluripotency, VEGF signaling, Molecular Mechanisms of Cancer, B Cell Receptor, PDGF, ERBB, Matrix Metalloproteases and PI3K/AKT signaling pathways involving transcripts detected in the integrative analysis (Figure 1C, Table 1).

**Table 1:** Top ranked canonical pathways identified by in silico analysis

Ingenuity Canonical Pathways (IPA)	Molecules(IPA)	*P*-value	KOBAS 2.0 Related Pathways	*P*-value
Human Embryonic Stem Cell Pluripotency	*FGFR1*,***TGFB3,PDGFRB,PDGFRA,NTF3*,***FGF2*,***PIK3R1,TCF7L1,NOG, TGFBR2*,***FZD3*,***NTRK2,FOXO1,S1PR1***	*P* < 0.001	Developmental Biology (Reactome)	0.031
VEGF Signaling	***NRAS,KDR,BCL2*,***ACTC1,FIGF,PRKC*, ***ROCK1,PIK3R1,ROCK2,FOXO1***	*P* < 0.001	Signaling by VEGF (Reactome)	0.009
Molecular Mechanisms of Cancer	*PLCB1*,***TGFB3,CDC25A,PIK3R1*,** *SMAD9,ADCY1*,***NRAS,TGFBR2*,** *ARHGEF17*,***CDKN1B,BCL2,RHOB*,** *PRKCB,FZD*,***CDK6,FOXO1,PMAIP1***	*P* < 0.001	Pathways in cancer (KEGG PATHWAY)	0.017
B Cell Receptor Signaling	***BCL6,NRAS,MAP3K12****,PRKCB*,***EGR1,PIK3R1,PPP3CA,FOXO1,MEF2C, CFL2***	*P* < 0.001	Signaling by the B Cell Receptor (BCR) (Reactome)	0.008
PDGF Signaling	***NRAS,PDGFRB,SRF,PDGFRA***,***PRKCB,PIK3R1***	0.003	Signaling by PDGF (Reactome)	0.003
ErbB Signaling	***NRAS,BTC,ERBB4****,PRKCB*,***PIK3R1, FOXO1***	0.004	Signaling by EGFR, ERBB2, ERBB4 (Reactome)	0.003
Matrix Metalloproteases	***RECK,MMP12,MMP1***	0.032	Extracellular matrix organization (Reactome)	0.018
PI3K/AKT Signaling	***NRAS,CDKN1B,BCL2,PIK3R1, FOXO1***	0.043	PI3K/AKT activation (Reactome)	0.003

The integrative analysis revealed 22 over- and 233 down-expressed genes which were used as input in IPA and KOBAS 2.0 software.

In bold: genes experimentally validated as regulated by miRNAs (miRtarbase).

### miRNA and mRNA validation

**Figure 2:**
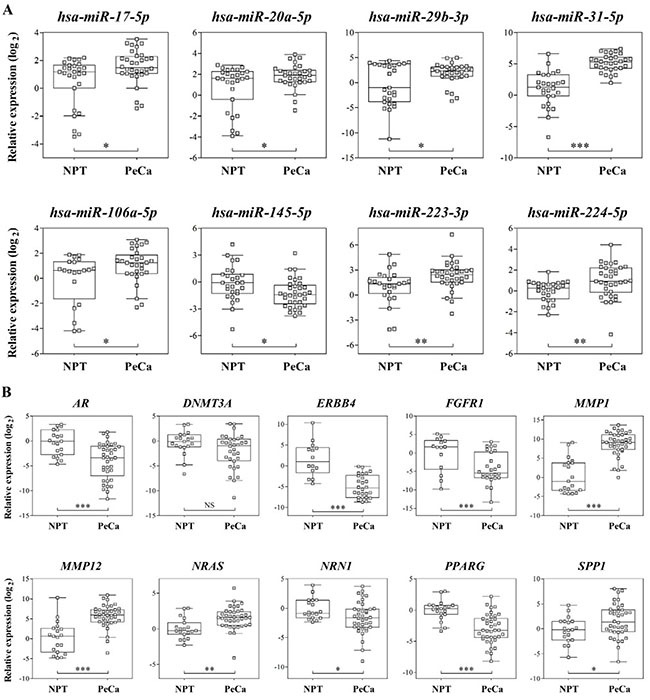
(**A**) Relative expression of eight selected miRNAs and ten (**B**) mRNAs by RT-qPCR in validation set of samples. *RNU48* (miRNAs) and *GUSB* (mRNAs) were used as references for RT-qPCR analysis. Parametric t test was applied to compare tumors with non-neoplastic penile tissue. NPT: Non-neoplastic penile tissue; PeCa: Penile Carcinoma, NS: not significant; *P < 0.05; **P < 0.01; ***P < 0.001.

Eight miRNAs and 10 transcripts were evaluated by RT-qPCR analysis in the same set of samples used for microarray and in an additional group of tumors (validation set) (Figure 2; Supplementary Table 3). The candidates for validation were selected according to the following criteria: (a) negative correlation between the mRNA and its miRNA regulator detected in the integrative analysis, (b) prognostic association, (c) high FC, (d) low FDR, and (e) low *P-value* in the miRNA and mRNA microarray analysis. Overexpression of *hsa-miR-20a-5p*, *hsa-miR-29b-3p, hsa-miR-31-5p, hsa-miR-224-5p, hsa-miR-106a-5p, hsa-miR-17-5p, hsa-miR-223-3p* and down-expression of *hsa-miR-145-5p* were confirmed by RT-qPCR analysis in the validation samples set (Figure 2A). All, with the exception of *hsa-miR-20a-5p* and *hsa-miR-29b-3p*, were also confirmed as significantly dysregulated in the same set of samples used for microarray (Supplementary Table 3).

Significant down-expression of *AR, ERBB4, FGFR1, NRN1* and *PPARG* and overexpression of *MMP1, MMP12, NRAS* and *SPP1* were confirmed by RT-qPCR assays in the validation set of samples (Figure 2B; Supplementary Table 3). Although not significant, *DNMT3A* presented decreased expression in the tumor samples.

In order to optimize the accuracy to distinguish tumors from non-neoplastic tissues, a higher discriminating power for the assessed markers was prioritized over the miRNA/mRNA interaction results. Three miRNAs and three mRNAs, presenting higher AUC (AUC for *hsa-miR-31-5p* = 0.861, *hsa-miR-224-5p* = 0.739 and *hsa-miR-*223-*3p* = 0.733, *MMP1* = 0.923, *MMP12* = 0.865 and *PPARG* = 0.851) were selected to construct a molecular classifier. miRNA and mRNA classifier accuracy was 79% and 89%, respectively. Sensitivity and specificity were 82% and 74% for miRNA classifier and 92% and 83% for mRNA classifier, respectively (Supplementary Figure 4).

### *MMP1* is a potential prognostic marker for lymph node metastasis in PeCa

**Figure 3:**
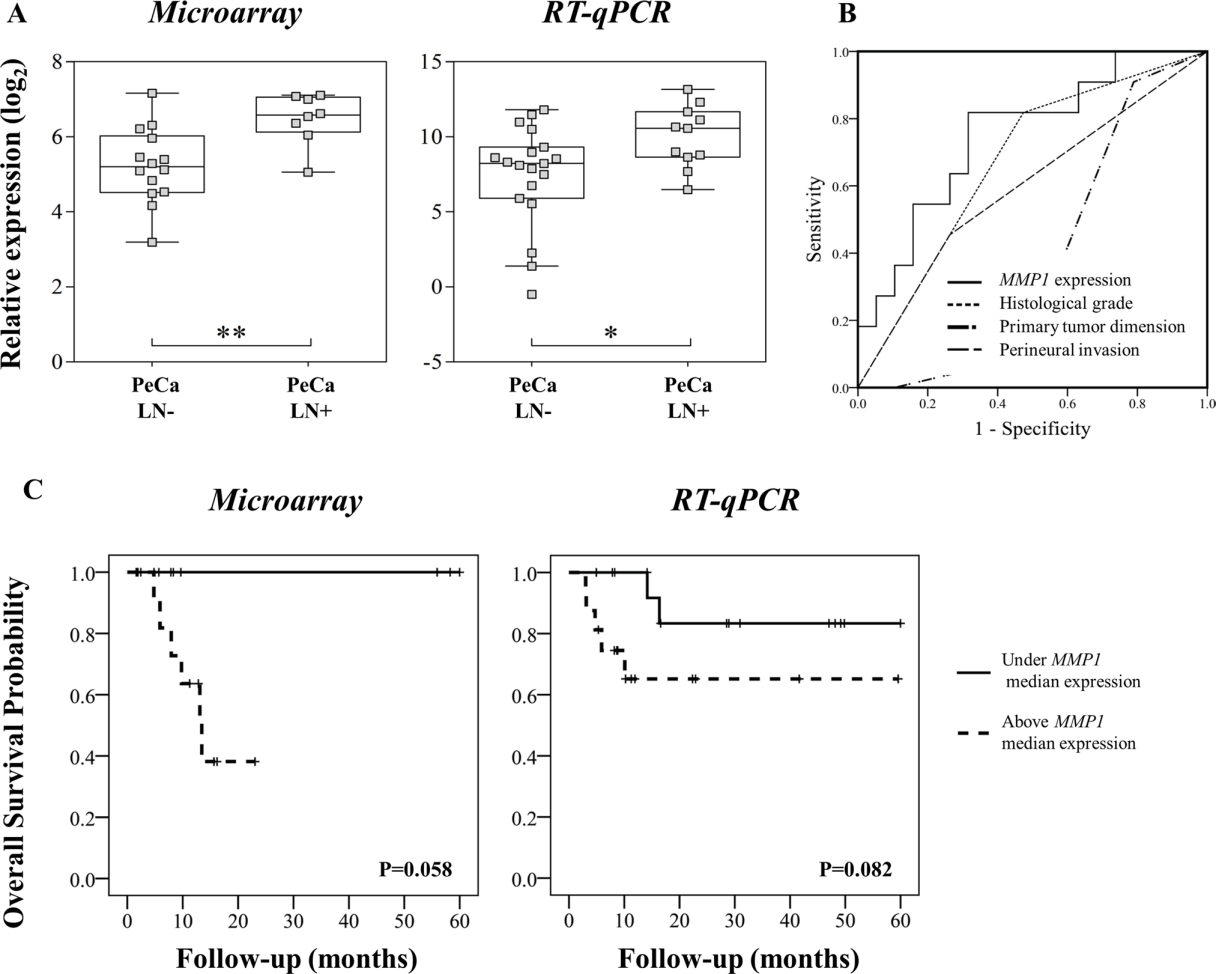
(**A**) Microarray and RT-qPCR data revealed higher *MMP1* expression in primary tumors from patients that presented inguinal lymph node metastasis (LN+). (**B**) *MMP1* was a better predictor of lymph node status compared with histological grade, primary tumor size (T1-T4) and perineural invasion. Area under the curve (AUC) for *MMP1* expression: 0.751, histological grade: 0.672; primary tumor size (T1-T4): 0.376 and perineural invasion: 0.596. (**C**) Overall survival analyses of PeCa patients according to *MMP1* expression patterns detected by microarray and RT-qPCR analyses. Kaplan-Meier curves show high expression of *MMP1* (defined as values above the median expression) associated with shorter survival.

The integrative analysis results were compared with clinical and pathological data including lymph node metastasis, tumor size and perineural invasion (Supplementary Table 4). Statistically significant associations were identified for lymph node metastasis (12 genes and three miRNAs), tumor size (eight genes and one miRNA) and perineural invasion (*UHMK1* gene) (*P* < 0.01, FDR < 20%). Among these genes, increased expression of *MMP1* was observed in cases with lymph node metastasis, further confirmed by RT-qPCR in the validation set of samples (Figure 3A).

*MMP1* expression was a better predictor factor of lymph node metastasis than well established pathological parameters (AUC: *MMP1* = 0.751; histological grade = 0.672; perineural invasion = 0.596; tumor size = 0.376) (Figure 3B, Supplementary Table 5). Higher *MMP1* expression levels (in both microarray and RT-qPCR analysis) were associated with shorter survival, although not significant (Figure 3C). The *hsa-miR-145*, which regulates *MMP1*, was down-expressed compared with NPT. However, no association with this miRNA and pathological characteristics was observed.

### Differentially expressed miRNA and mRNA according to HPV status

Seven of 23 PeCa samples (30.4%) presented high-risk HPV genotype (6 HPV16 cases and 1 HPV18). The mRNA hierarchical clustering analysis revealed four of seven HPV-positive cases grouped together in cluster 1. In addition, the miRNA analysis presented the cluster 2 enriched with five of seven HPV-positive tumors. The cluster 3, which comprised patients with poor prognosis, showed a limited number of HPV-positive cases (2 for mRNA and 1 for miRNA cluster analysis) (Figure 1A and 1B).

The integrative analysis revealed five miRNAs (*hsa-let-7b-5p, hsa-miR-146a-5p, hsa-miR-185-5p, hsa-miR-29b-3p* and *hsa-miR-505-3p*) down-expressed in HPV positive cases compared with negative cases. Thirteen transcripts (down-expression of *CSF1* and *PKD2* and overexpression of *PPM1B, INPP5A, LONRF1, WASF3, PRKG1, NTF3, NBEA, EGR1, RGS5, NTRK2* and *OLFM1*) were also detected as dysregulated in HPV positive cases (FDR > 20%) (Supplementary Table 4).

## DISCUSSION

Dysregulation of the transcriptome and miRNA machinery is a common process in cancer development and progression. In the present study, gene expression and miRNA profiles were able to distinguish one cluster (cluster 3), which contained the majority of cases presenting aggressive clinicopathological features (lymph node metastasis and T3-T4 tumor size).

Integrative analysis of miRNA and mRNA is an important tool to identify potential diagnostic and prognostic markers in a variety of tumors [23, 24]. Recently, Zhang *et al*. [22] described 384 differentially expressed miRNAs with potential involvement in penile malignant transformation. Comparison of the differentially expressed miRNAs found in the present study with those described by Zhang *et al*. [22] revealed a 15% overlap. A plausible explanation for this is the use of different methods in the two studies (next generation sequencing of a pool of 10 samples *versus* the Taqman Low density array with a larger number of cases used in our study).

Using *in silico* molecular analysis, we described 598 interactions involving 68 miRNAs and 255 mRNAs. *hsa-miRNA-31-5p* was identified as having the highest fold change (FC = 352.4) in tumor samples, as previously described in several epithelial tumors (head and neck squamous cell, colorectal, prostate and lung cancer) [26, 27], as well as in premalignant colonic lesions [27]. In addition, this miRNA in combination with *hsa-miR-224-5p* and *hsa-miR-223-3p* were able to distinguish tumors from NPT, indicating its importance in PeCa development. A variety of experimentally validated target genes regulated by *hsa-miR-31-5p* has been described in different tumor types [26], including *AR* and *DNMT3A* genes. The androgen receptor (AR) is a critical transcription factor with prognostic value in breast cancer and prostate [28, 29] as well as in bladder carcinomas [30].

The expression of *AR* was significantly decreased in penile tumors, both in the microarray and in the set of cases used for validation (*p* < 0.001). Interestingly, the *ESR1* mRNA was also down-expressed in PeCa, with several miRNAs that potentially regulate this gene being overexpressed (Supplementary Table 2). In addition, *AR* and *ESR1* presented the highest number of predicted functional interactions with other genes (Figure 1C), suggesting their potential as molecular drivers in PeCa. Functional loss of the *AR* gene by promoter hypermethylation has been described as involved in PeCa development [31]. A previous study by our group on penile tumors did not reveal *AR* hypermethylation [18]. In addition to hypermethylation, dysregulation of the miRNAs that target *AR* may also contribute to PeCa development and progression. We found four overexpressed miRNAs predicted as involved in the regulation of the *AR* gene (*hsa-miR-31-5p, hsa-miR-34a-5p*, *hsa-miR-205-5p* and *hsa-miR-185-5p*) and possibly associated with its down-expression.

Potential dysregulation of the VEGF signaling pathway was also observed by *in silico* analysis. Genes involved in this pathway have already been shown to be regulated by miRNAs [32]. The *hsa-miR-424-5p* overexpression, which has previously been associated with angiogenesis [33], was described in our study. This miRNA regulates the *FGFR1* gene, confirmed by RT-qPCR as down-expressed. A clinical trial to test anti-angiogenic therapy in PeCa patients (NCT02279576) started in 2014. Overall, our findings give additional support to the relevance of genes and miRNAs associated with angiogenesis in PeCa.

While the role of VEGF and angiogenesis remains unexplored in PeCa, the involvement of the EGF pathway has been reported. Recently, *EGFR* gene mutation, amplification and overexpression have been described in PeCa, suggesting the potential of using therapeutic strategies targeting EGFR in a selected group of patients [34, 35]. Currently, EGFR-targeted therapies are found to be clinically useful in PeCa patients (approximately 30 cases) [34–39]. Although the *EGFR* expression levels were not identified in this study, four ligands of *EGFR* (*AREG*, *EREG, TGFA* and *EPGN*) were overexpressed, as well as the downstream effector *NRAS*. These data suggest that this pathway is dysregulated and, hence contribute to penile carcinogenesis.

Previously, we have shown the association of *PPARG* loss with poor prognostic features in PeCa (advanced clinical and T stage and lymph node metastasis) [12]. We have also demonstrated that *PPARG* loss leads to its down-expression as a result of a gene dosage effect. Increasing *PPARG* activation using agonists has been shown as a powerful strategy to either inhibit cell proliferation or induce apoptosis [40]. The use of *PPARG* gene as a therapeutic target is already being applied in a considerable number of tumors, including breast, prostate, colorectal and thyroid [40].

The metalloprotease genes family was shown to have the highest fold-changes, including *MMP1* (FC = 51.1)*, MMP10* (FC = 52.5) and *MMP12* (FC = 89.9). In the validation set of PeCa samples, overexpression of *MMP1* and *MMP12* was confirmed by RT-qPCR. In addition, a classifier with *MMP1* and *MMP12* in combination with *PPARG* was able to discriminate PeCa from NPT. Molecular alterations have been reported as preceding morphological and pathological changes [41, 42]. Therefore, markers that could indicate regions with genetic alteration may be of great value to define margin assessment. Along with the fact that matrix metalloproteinase was one of the most important pathways dysregulated in PeCa, these results suggest that MMPs genes might act as oncogenic drivers for PeCa development.

Integrative analysis revealed that *MMP1* and *MMP12* may be regulated by *hsa-miR-145-5p*, which was down-expressed in PeCa. Similarly, Zhang *et al*. [22] reported down-expression of this miRNA in 10 PeCa. Down-expression of *hsa-miR-145* has been described in several cancer types, mediating suppression of cell growth, invasion and metastasis [43]. In prostate cancer, *hsa-miR-145* down-expression has been reported as a predictor of poor prognosis [44].

Although *hsa-miR-145* down-expression was unable to predict poor prognosis in our cohort of cases, its target *MMP1* showed increased expression levels in patients with lymph node metastasis. *MMP1* gene expression levels were a better predictor of lymph node metastasis than tumor size, histological grade and perineural invasion. In agreement with this finding, Zheng *et al*. [45] showed that *hsa-miR-145* overexpression altered *MMP1* and *MMP9* mRNA and protein levels, with subsequent inhibition of invasion, metastasis and angiogenesis in gastric cancer cells. Although some prognostic molecular makers have been reported in PeCa, the stratification of the patients is currently based on clinical and histopathological features [11, 31, 39, 46, 47]. In the present study, *MMP1* showed superior performance in discriminate lymph node metastasis in PeCa compared with established clinical-pathological parameters. The Supplementary Table 4 shows a list of transcripts and miRNA potentially associated with prognosis and clinical features in PeCa (*P* < 0.01 and FDR < 20%). However, none of them were confirmed as predictors of lymph node metastasis (data not shown).

Although controversial, HPV infection has been reported as a risk factor involved in the PeCa etiology influencing the prognosis [5]. Gregoire *et al*. [48] reported an association between HPV positive infection and poor prognosis in penile cancer patients. However, HPV infection was also reported as conferring a survival advantage in these patients [49, 50]. Lont *et al*. [50] reported a 5-year cancer-specific survival rate of 93% for HPV-positive and 78% for HPV-negative cases. The cluster 3 (enriched with cases showing poor prognosis features) of our miRNA and mRNA profiles revealed a limited number of HPV positive cases, which suggests a less aggressive phenotype. In addition, a set of five miRNAs was down-expressed in HPV positive cases, including the *hsa-let-7b-5p, hsa-miR-146a-5p, hsa-miR-185-5p, hsa-miR-29b-3p* and *hsa-miR-505-3p*. Recently, Peta *et al*. [21] reported lower expression levels of *hsa-miR-146a* in high-risk HPV positive than in negative tumors.

In conclusion, our integrative analysis was able to identify miRNA and mRNA related with cancer development and progression. Furthermore, *MMP1* is a predictive marker of lymph node metastasis in PeCa. We also pinpointed PPARG, VEGF, EGFR and matrix metalloproteinase pathways as dysregulated in PeCa samples, endorsing their involvement as potential targets for PeCa treatment.

## MATERIALS AND METHODS

### Patients and sample collection

A total of 101 samples was included in this study: 59 penile carcinomas (PeCa), 26 surrounding normal tissues (SNT) and 16 normal glans (NG) (Supplementary Figure 5). Fresh frozen PeCa tissue and SNT were obtained from untreated patients who underwent tumor resection at A.C. Camargo Cancer Center, São Paulo, Barretos Cancer Hospital, Barretos, and Faculty of Medicine, Botucatu, SP, Brazil. Normal glans samples were obtained from autopsies. The majority of the penile cancer tissue samples were confirmed histologically as usual penile carcinomas. All samples used in this study, including SNT, were submitted to cellular macrodissection and histology confirmation. Tumor samples presented at least 80% of tumor cells and SNT were composed by normal epithelial cells. Written informed consent was obtained from all patients or relatives. The Human Research Ethic Committees of the aforementioned Institutions approved the study (Protocols #1230/09: A.C. Camargo Cancer Center; #363–2010: Barretos Cancer Hospital, and #501.229/2013: Faculty of Medicine, Botucatu, SP, Brazil).

Previously, we reported a transcriptomic analysis (4 × 44K, Agilent Technologies, Santa Clara, CA, USA) of 33 PeCa and a pool of four NG [18]. Twenty-three PeCa samples had tissue available for miRNA expression analysis. Seven SNT (paired with 7 PeCa) and five NG samples were also included in the miRNA expression analysis as control. Integrative analysis of mRNA and miRNA expression data was performed for 23 PeCa samples.

Quantitative RT-PCR was applied in the same group of samples used in the array experiments (21 PeCa, 6 SNT and 5 NG for miRNA microarray analysis and 20 PeCa and 3 NG for mRNA microarray analysis) and in the validation set of samples (33 PeCa, 20 SNT and 7 NG for miRNA and 36 PeCa, 9 SNT and 10 NG for mRNA analysis). Non-neoplastic penile tissues (NPT) composed by SNT and NG samples were compared with tumor tissue. Clinical and histopathological data for the PeCa samples are shown in Table 2.

**Table 2:** Clinical and histopathological features of PeCa cases (N = 59)

Variable	Samples used for microarray *N* (%)	Validation set of samples *N* (%)
*Number*	23	36
*Age (years)*		
Median (range)	59.2 (31–91)	58.6 (30–92)
*Follow-up (months)*		
Median (range)	15.3 (1.6–60)	20.3 (1–60)
*Histological classification*		
Usual SCC	20 (87%)	33 (91.7%)
Mixed*	1 (4.3%)	3 (8.3%)
Papillary	2 (8.7%)	0 (0.0%)
*Histological grade*		
I	5 (21.7%)	12 (33.3%)
II	9 (39.2%)	17 (47.2%)
III	7 (30.4%)	7 (19.5%)
NI	2 (8.7%)	0 (0.0%)
*HPV infection*		
HPV-Positive#	7 (30.4%)	9 (25%)
HPV-Negative	16 (69.6)	27 (75%)
*Lymph node metastasis*		
Presence	9 (39.2%)	13 (36.1%)
Absence	13 (56.5%)	21 (58.3%)
ND	1 (4.3%)	2 (5.6%)
*Perineural Invasion*		
Presence	5 (21.7%)	13 (36.1%)
Absence	18 (78.3%)	23 (63.9%)
*T Stage*		
1–2	14 (60.8%)	22 (61.1%)
3–4	9 (39.2%)	14 (38.9%)

Patients were divided in two groups, the same set of samples used in the microarray experiments (*N* = 23) and a validation set of samples (*N* = 36).

SCC: squamous cell carcinoma; HPV: human papilloma virus; ND: Not determined. *Mixed tumors comprised: 3 usual - sarcomatoid subtype and one usual - papillary subtypes. NI: Not informed (two papillary carcinomas). ^#^Sixteen cases were HPV positive: seven cases were evaluated by microarrays (6 HPV16 and 1 HPV18) and nine for data validation (8 HPV16 and 1 HPV33).

Human papilloma virus (HPV) status was investigated for all PeCa samples (Linear Array HPV Test Genotyping, Roche Molecular Diagnostics, Branchburg, NJ, USA). HPV-positive cases were detected in seven of the 23 cases (30.4%) used for microarray analysis and in nine of the 36 PeCa (25%) in the validation set (Table 2).

### miRNA and mRNA profiles in PeCa

Total RNA was obtained from macrodissected fresh frozen tissues using miRNAeasy Kit (Qiagen, Venlo, Limburg, Netherlands). TaqMan Human MicroRNA Assay System Set v2.0 (Applied Biosystems, Foster City, CA, USA) was used for miRNA expression analysis. A set of pre-defined primers (Megaplex RT primers™, Pool A, Applied Biosystems, Foster City, CA, USA) was used for cDNA synthesis as recommended by the manufacturer. Data was normalized using the Pfaffl model [51] with *MammU6*, *RNU44* and *RNU48* as references. Low abundant miRNAs (undetermined quantification cycle) that were observed in more than 20% of samples from each comparison were excluded from the analysis. Biological groups were compared using the two-sample *t-test* with BRB ArrayTools software (v. 4.4.0) [52], establishing a two-tailed *P-value* of < 0.01 with a low false discovery rate ratio (FDR< 5%) and at least two fold changes (FC > 2) as significant.

mRNA expression analysis was performed using the Whole Human Genome 4×44K microarray platform (Agilent Technologies) as described by Kuasne *et al*. [18] Transcriptomic data are available on the Gene Expression Omnibus (GEO) database (GSE57955). Unsupervised hierarchical clustering analysis of miRNA and mRNA expression was accomplished using one minus correlation metric and complete linkage. The Kaplan Meier curve and log rank test were undertaken to estimate the overall survival [53].

### Integrative analysis

Integrative analysis using miRNA and mRNA expression data from 23 tumor samples was based on predicted and experimentally validated miRNA/mRNA interactions. Predicted miRNA/mRNA interactions were performed using miRWalk 2.0 [54], in at least 10 of 12 target prediction tools (Last access: October 2015). In addition, experimentally validated miRNA/mRNA interactions by reporter assays were obtained from miRTarBase database [55] (Last access: October 2015). Subsequently, negative miRNA/target mRNA correlation (r < 0) presenting inverted FC was considered.

Transcripts regulated by miRNAs were submitted to *in silico* analysis using Ingenuity Pathways Analysis (IPA, Ingenuity^®^ System) and KEGG Orthology Based Annotation System (KOBAS 2.0) software [56]. Protein-protein interactions were assessed with known and predicted physical interactions using I2D version 2.0 (http://ophid.utoronto.ca/i2d). The resulting networks were visualized using NAViGaTOR version 2.3 [57]. http://ophid.utoronto.ca/navigator. Clinical associations were performed using two-sample *t-test* (*P <* 0.01, FDR < 20%).

### Evaluation of differentially expressed miRNAs and mRNA by reverse transcription quantitative PCR (RT-qPCR)

Eight miRNAs (*hsa-miR-20a-5p, hsa-miR-29b-3p, hsa-miR-31-5p, hsa-miR-224-5p, hsa-miR-106a-5p, hsa-miR-17-5p, hsa-miR-223-3p*, and *hsa-miR-145-5p)* and ten mRNAs (*AR, DNMT3A, ERBB4, FGFR1, MMP1, MMP12, NRAS, NRN1, PPARG*, and *SPP1)* were evaluated by RT-qPCR. The candidates for validation were selected according to the following criteria (in descending order of importance): (a) miRNA-mRNA pair detected in the integrative analysis with negative correlation, (b) prognostic association, (c) high FC, (d) low FDR, and (e) low *P-value* in the individual miRNA and mRNA analysis.

The RT-qPCR experiments followed the MIQE guideline recommendations [58]. *RNU48* and *GUSB* were used as references for miRNAs and mRNA, respectively, as previously reported [19]. cDNA synthesis was performed using total RNA and the TaqMan miRNA Reverse Transcription Kit (Applied Biosystems, Foster City, CA, USA), according to the manufacturer's recommendations. MicroRNA expression was assessed using the TaqMan^®^ MicroRNA Assay (Applied Biosystems, Foster City, CA, USA). For mRNA expression analysis, cDNA synthesis was conducted as previously described [59]. The reactions were carried out by automated pipetting (QIAgility, Qiagen, Courtaboeuf, France) in duplicate using TaqMan^®^ Universal PCR Master Mix, No AmpErase^®^ UNG (Applied Biosystems, Foster City, CA, USA) (miRNA) or Syber Green Master Mix (mRNA) using the 7900 Real time PCR System (Applied Biosystems, Foster City, CA).

Relative quantification of the expression levels was calculated according to Pfaffl method [51]. Parametric t test was applied to compare tumors with non-neoplastic penile tissue (NPT) (comprising SNT and NG samples) and according to clinicopathological features. Statistical analysis was performed using GraphPad Prism5 (San Diego, CA, USA) and SPSS version 21.0 (SPSS, Chicago, IL, USA).

Classifiers were designed to distinguish PeCa from NPT, using the markers presenting the higher area under the ROC (Receiver Operating Characteristic) curve (AUC). The support vector machine (SVM) method was applied and performance was assessed by leave-one-out-cross-validation (LOOCV) using BRB ArrayTools software (v. 4.4.0). A similar approach was carried out to predict lymph node status. Overall survival analysis was performed using Kaplan-Meier and log rank test. In this analysis, gene expression was dichotomized as low (bellow median) and high (above median) expression.

## SUPPLEMENTARY MATERIALS FIGURES AND TABLES






