# Supplementary material for: Integrative miRNA and mRNA analysis in penile carcinomas reveals markers and pathways with potential clinical impact
Source: Oncotarget. 2017 Jan 21;8(9):15294–306. doi: 10.18632/oncotarget.14783 (PMC5362487; doi:10.18632/oncotarget.14783)
Supplement: Supplementary file 1 [file oncotarget-08-15294-s001.pdf]

Integrative miRNA and mRNA analysis in penile carcinomas reveals markers and pathways with potential clinical impact

Supplementary Materials

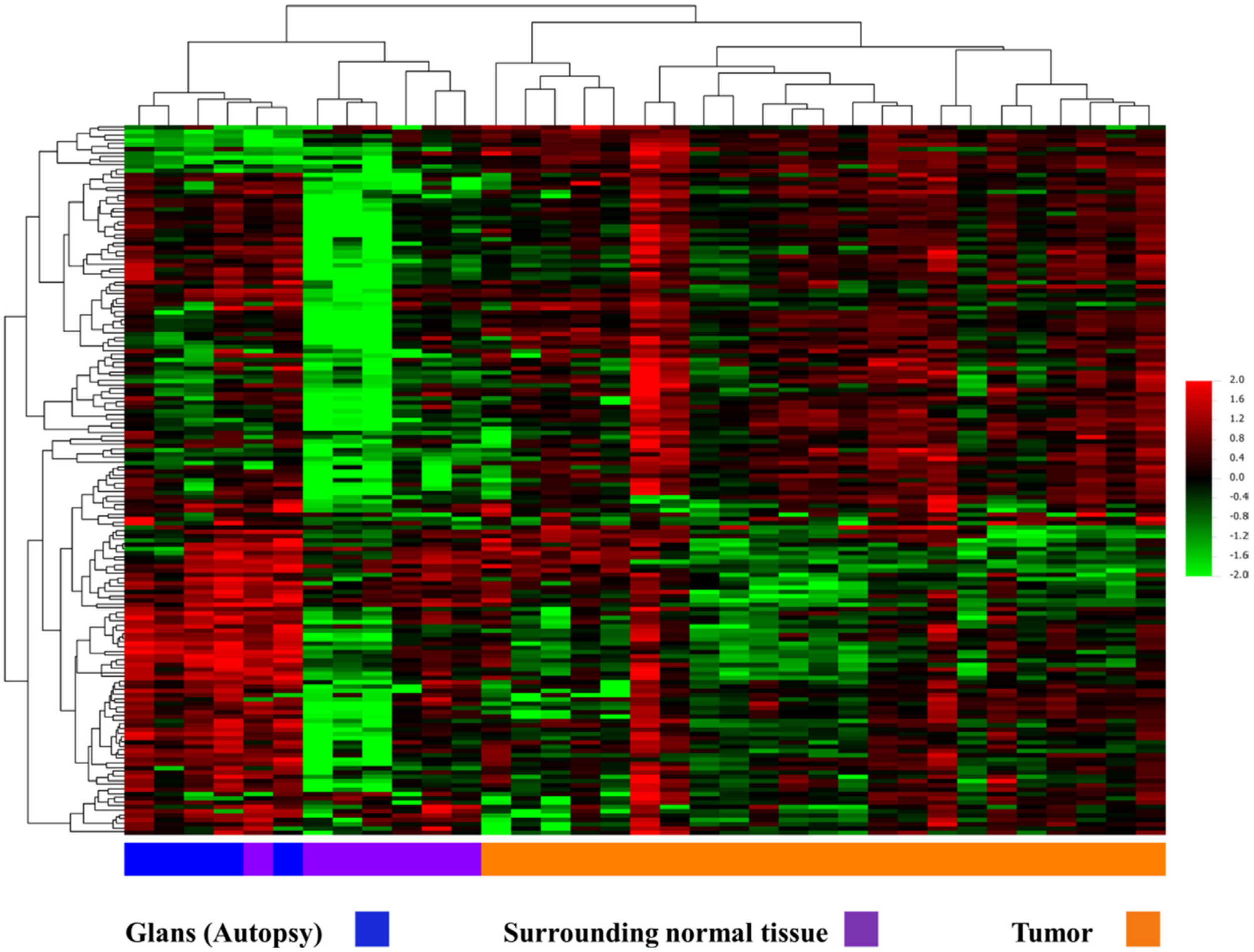

Supplementary Figure 1: Unsupervised hierarchical clustering analysis of miRNAs revealed two main clusters, one with PeCa samples and the second composed by non-neoplastic penile tissues (glans samples and surrounding normal tissues). 165 miRNAs (SD > 1.0; Metric: 1 minus correlation; Linkage: complete).

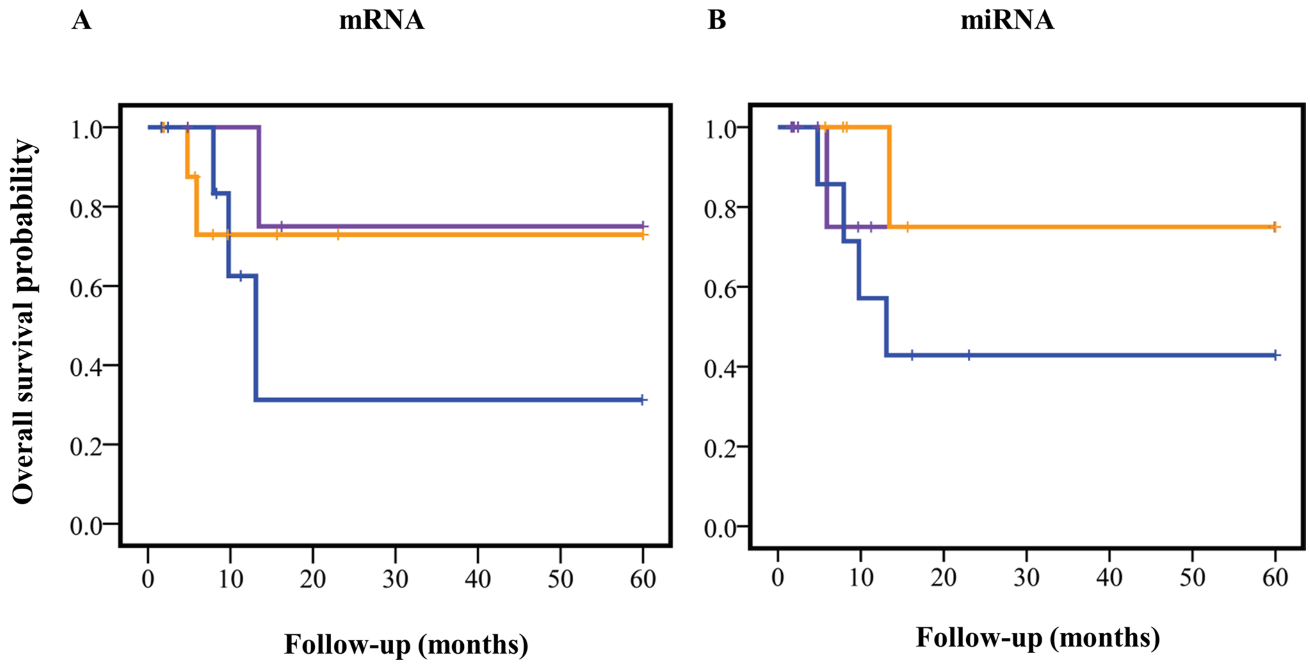

Supplementary Figure 2: Kaplan-Meier curves representing the three main clusters of PeCa samples according to mRNA and miRNA analyses. Gene expression and miRNA profiles were able to distinguish patients according to aggressive features. The cluster 3 (blue line) found in mRNA (**A**) and miRNA (**B**) hierarchical clustering analysis were enriched with cases with poor prognosis features (lymph node metastasis, T3-T4 tumor stages, and perineural invasion) and presented shorter overall survival (non-significant– mRNA ($P = 0.49$) and miRNA ($P = 0.39$).

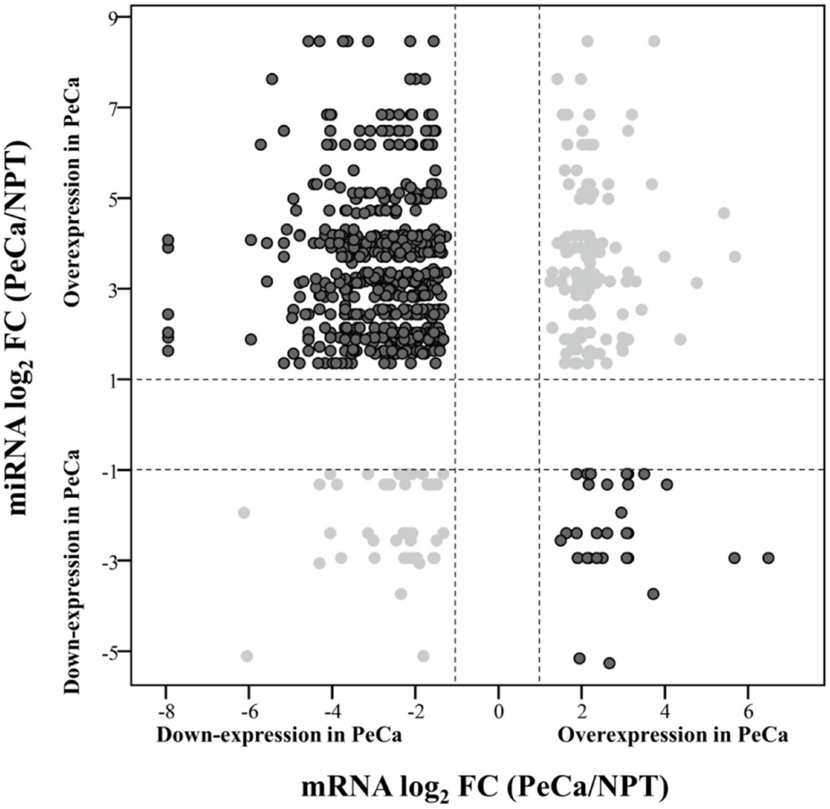

Supplementary Figure 3: Scatterplot showing the $\log_2$ values of miRNA and mRNA fold changes (FC) by comparing penile carcinomas (PeCa) and non-neoplastic penile tissues (NPT). Dark gray: 598 interactions with the expected direction (79%). Light gray: 157 interactions with unexpected directions (21%).

A

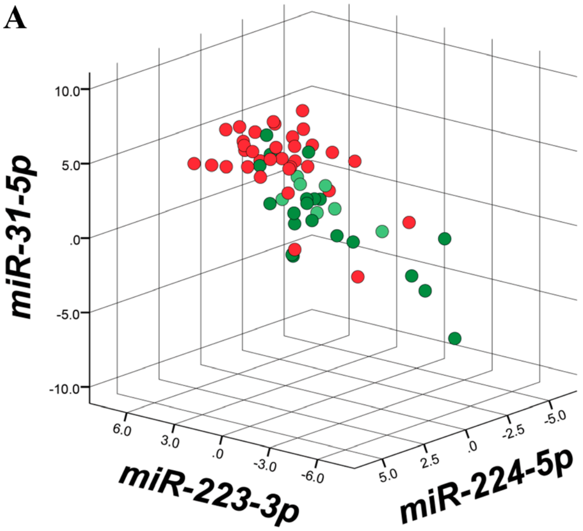

B

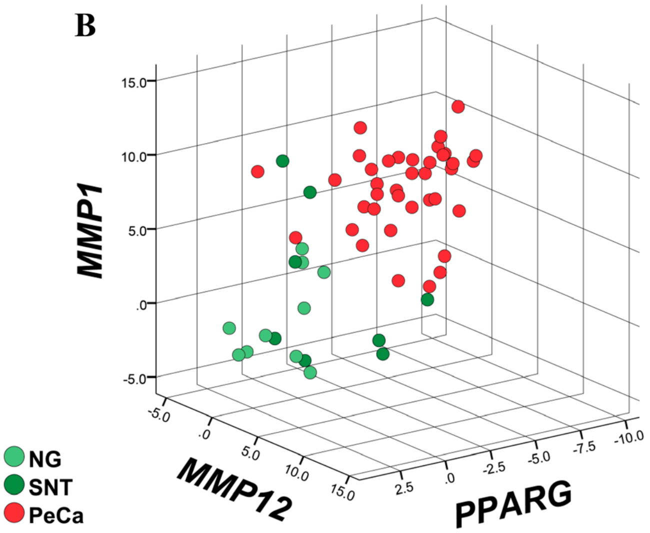

C

PERFORMANCE SVM (LOOCV)		3-miRNAs Algorithm	
		Non-tumor	Malignant
**Histology**	NG	4	3
	SNT	16	4
	PeCa	6	28
**Sens (CI 95%)**		82% (65-93)	
**Spec (CI 95%)**		74% (54-89)	
**PPV (CI 95%)**		80% (63-92)	
**NPV (CI 95%)**		77% (56-91)	

D

PERFORMANCE SVM (LOOCV)		3-mRNAs Algorithm	
		Non-tumor	Malignant
**Histology**	NG	10	0
	SNT	5	3
	PeCa	3	35
**Sens (CI 95%)**		92% (79-98)	
**Spec (CI 95%)**		83% (59-96)	
**PPV (CI 95%)**		92% (79-98)	
**NPV (CI 95%)**		83% (59-96)	

Supplementary Figure 4: (**A**) Graph plot showing the relative expression of the combination of (A) *hsa-miRNA-31-5p*, *hsa-miRNA-223-3p* and *hsa-miRNA-224-3p* and **(B)** *MMP1*, *MMP12* and *PPARG*. The classification performance involving these three miRNAs (**C**) and three genes (**D**) reveal high sensitivity and specificity in discriminate tumor from non-tumor samples. The AUC values were: *hsa-miR-31-5p* = 0.861, *hsa-miR-224-5p* = 0.739, *hsa-miR-223-3p* = 0.733, *MMP1* = 0.923, *MMP12* = 0.865 and *PPARG* = 0.851. PPV: positive predictive value; NPV: negative predictive value. NG: normal glans, SNT: surrounding normal tissue, PeCa: penile carcinoma, SVM: Support vector machine, LOOCV: Leave-one-out-cross-validation, Sens: sensitivity, Spec: specificity, PPV: positive predictive value; NPV: negative predictive value.

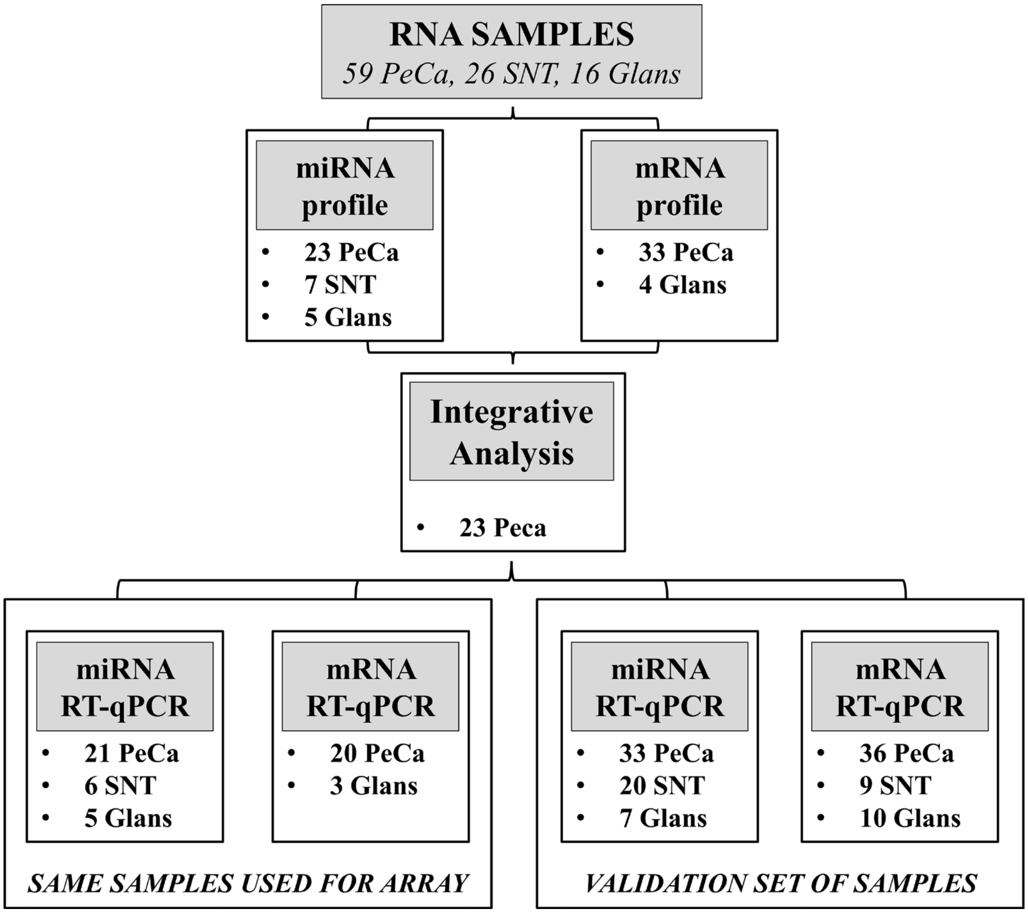

Supplementary Figure 5: A total of 101 samples were obtained: 59 PeCa, 26 SNT and 16 glans. Twenty-three PeCa samples were used for miRNA and mRNA integrative analysis. RT-qPCR (Ten mRNAs and eight miRNAs) were performed in the same set of samples used for microarray and in a validation set of samples. PeCa: Penile Cancer; SNT (surrounding non-malignant tissue).

Supplementary Table 1: Eight-one differentially expressed miRNA found in the comparison between PeCa (*N* = 23) and non-neoplastic penile tissue samples (seven SNT and five normal glans). See Supplementary_Table_1

Supplementary Table 2: The integrative analysis resulted in 598 interactions with negative correlation involving 68 miRNAs and 255 mRNA (miRWalk2.0 and/or miRTarBase). In bold, mRNA and miRNA experimentally validated (miRtarbase). See Supplementary_Table_2

Supplementary Table 3: Expression patterns of genes and miRNAs selected for evaluation by RT-qPCR in the same set of samples used in the array experiments and in an independent set of samples

Gene	Selection criteria	Same set of samples used for microarray (*N* = 20 PeCa and 3 NPT)		Validation set of samples (*N* = 36 PeCa and 19 NPT)	
		FC	*P*#	FC	*P*
AR	Low FC and highest number of interactions with other genes in miRNA-mRNA network (Figure 1C) - Integrative analysis (predicted to be regulated by *miR-31-5p*)	–6.0	0.131	–13.1	**< 0.001**
DNMT3A	Integrative analysis (predicted to be regulated by *miR-29b-3p* and *miR-31-5p*)	–10.8	0.025	–3.1	0.071
ERBB4	Top canonical pathways–integrative analysis	–151.6	< 0.001	–96.1	**< 0.001**
FGFR1	Top canonical pathways–integrative analysis	–6.7	0.136	–16.5	**0.008**
MMP1	Top canonical pathways–integrative analysis (predicted to be regulated by *miR-145-5p*) and high FC	930.7	0.053	304.6	**< 0.001**
MMP12	Top canonical pathways–integrative analysis (predicted to be regulated by *miR-145-5p*) and high FC	1454.0	< 0.001	41.2	**< 0.001**
NRAS	Top canonical pathways (6 pathways)–integrative analysis (predicted to be regulated by *miR-145-5p*)	2.4	0.013	2.8	**0.001**
NRN1	Integrative analysis	–3.9	0.118	–2.7	**0.021**
PPARG	Integrative analysis (predicted to be regulated by *miR-20a-5p*) and previous data**	–7.5	< 0.001	–8.2	**< 0.001**
SPP1	Previous data **	3.9	0.416	3.7	**0.035**
**miRNA**		**(*N* = 21 PeCa and 11 NPT)**		**(*N* = 33 PeCa and 27 NPT)**	
hsa-miR-20a-5p	Integrative analysis- predicted to regulate *PPARG*	2.2	0.467	2.0	**0.032**
hsa-miR-29b-3p	Integrative analysis- predicted to regulate *DNMT3A* and *ESR1*	3.8	0.320	4.6	**0.019**
hsa-miR-31-5p	Highest FC	119.4	**< 0.001**	17.7	**< 0.001**
hsa-miR-224-5p	Discriminates PeCa from NPT	3.1	**0.008**	1.9	**0.006**
hsa-miR-106a-5p	Associated with prognosis (Supplementary Table 4, lymph node metastasis)	3.8	**0.015**	2.2	**0.024**
hsa-miR-17-5p	Associated with prognosis (Supplementary Table 4, lymph node metastasis)	8.3	**< 0.001**	2.1	**0.010**
hsa-miR-223-3p	Discriminates PeCa from NPT	13.4	**< 0.001**	2.5	**0.009**
hsa-miR-145-5p	Integrative analysis–predicted to regulate *MMP12*, *MMP1* and *NRAS*	–4.9	**0.007**	-2.2	**0.018**

PeCa: Penile Carcinoma; NPT: non-neoplastic penile tissue; bold: $P < 0.05$. #No significant p value in the same set of samples used in the array experiments possibly due to the small number of normal samples used. However, the fold change (FC) was in the expected direction.
**Busso-Lopes et al. Genomic profiling of human penile carcinoma predicts worse prognosis and survival. *Cancer Prev Res (Phila)* 2015; 8(2):149-56.[12]
**Kuasne H et al. Genome-wide methylation and transcriptome analysis in penile carcinoma: uncovering new molecular markers. *Clin Epigenetics* 2015;7: 46.[18]

Supplementary Table 4: Dysregulation of miRNAs and mRNAs (integrative analysis) compared with pathological characteristics

Features	Gene	FC	*p*-value	FDR (%)	miRNA	FC	*p*-value	FDR (%)
Lymph node metastasis (positive *versus* negative)	*CLU*	3.7	0.001	9.3	*hsa-miR-106a-5p*	–2.1	0.005	15.9
	PKDCC	3.3	0.001	9.3	*hsa-miR-17-5p*	–2.1	0.005	15.9
	MMP1	2.4	0.005	15.9	*hsa-miR-512-3p*	–4	0.006	15.9
	AMIGO1	2.3	0.001	9.3				
	NTN4	2.2	0.008	15.9				
	FIGF	2	0.008	15.9				
	NFIA	1.9	0.004	15.9				
	UHMK1	1.6	0.007	15.9				
	ZDHHC17	1.5	0.006	15.9				
	CDC25A	–2.4	0.007	15.9				
	PMAIP1	–4.1	0.001	9.3				
	HMGA2	–4.7	0.007	15.9				
Tumor size (T3-T4 *versus* T2-T1)	*CLU*	3.4	0.001	11.8	*hsa-miR-320a*	–2.3	0.005	18.4
	PKDCC	3.3	0.001	11.7				
	SIX1	2.9	0.002	11.8				
	NFIB	1.9	0.004	15.7				
	ARHGEF17	1.8	0.001	11.7				
	EZH1	1.7	0.004	15.7				
	ZDHHC17	1.6	0.001	11.7				
	NRAS	–1.9	0.003	15.7				
Perineural invasion (positive *versus* negative)	*UHMK1*	2	0.001	4.7				
HPV status (positive *versus* negative)	*CSF1*	–1.7	0.044	> 20	*hsa-miR-505-3p*	–4.6	0.008	> 20
	PKD2	–1.6	0.041	> 20	*hsa-miR-29b-3p*	–3.1	0.046	> 20
	PPM1B	1.4	0.018	> 20	*hsa-miR-146a-5p*	–2.8	0.026	> 20
	INPP5A	1.5	0.02	> 20	*hsa-miR-185-5p*	–2.6	0.042	> 20
	LONRF1	1.6	0.009	> 20	*hsa-let-7b-5p*	–2.1	0.043	> 20
	WASF3	1.6	0.024	> 20				
	PRKG1	1.9	0.016	> 20				
	NTF3	1.9	0.004	> 20				
	NBEA	2.1	0.035	> 20				
	EGR1	2.3	0.023	> 20				
	RGS5	2.4	0.033	> 20				
	NTRK2	2.6	0.047	> 20				
	OLFM1	4	0.003	> 20				

Supplementary Table 5: *MMP1* expression performance to discriminate cases according to lymph nodes involvement.

Metrics	Microarray	RT-qPCR
**Sensitivity (95% CI)**	87.5% (47.4-99.7)	81.8% (48.2-97.7)
**Specificity (95% CI)**	71.4% (41.9-91.6)	68.4% (43.5-87.4)
**PPV (95% CI)**	63.6% (30.8-89.1)	60.0% (32.3-93.7)
**NPV (95% CI)**	90.9% (58.7-99.8)	86.7% (59.5-98.3)

PPV: positive predictive value; NPV: negative predictive value; CI: confidential interval
