# Supplementary material for: Integrative miRNA and mRNA analysis in penile carcinomas reveals markers and pathways with potential clinical impact
Source: Oncotarget. 2017 Jan 21;8(9):15294–306. doi: 10.18632/oncotarget.14783 (PMC5362487; doi:10.18632/oncotarget.14783)
Supplement: Supplementary file 2 [file oncotarget-08-15294-s002.docx]

**Table S1.**  Eight-one differentially expressed miRNA found in the comparison between PeCa (N=23) and non-neoplastic penile tissue samples (seven SNT and five normal glans).

| **miRNA**  **(miRBase ID)** | **assay ID** | **FC** | **P value** | **FDR** |
| --- | --- | --- | --- | --- |
| *hsa-miR-31-5p* | 2279 | 352.4 | <0.0000001 | <0.1% |
| *hsa-miR-135b-5p* | 2261 | 197.5 | <0.0000001 | <0.1% |
| *hsa-miR-141-3p* | 463 | 114.7 | <0.0000001 | <0.1% |
| *hsa-miR-429* | 1024 | 89.5 | <0.0000001 | <0.1% |
| *hsa-miR-200a-3p* | 502 | 72.5 | <0.0000001 | <0.1% |
| *hsa-miR-363-3p* | 1271 | 48.9 | 0.0031024 | 0.01 |
| *hsa-miR-455-5p* | 1280 | 39.6 | 0.0000002 | <0.1% |
| *hsa-miR-203a-3p* | 507 | 39.6 | 0.0000028 | <0.1% |
| *hsa-miR-455-3p* | 2244 | 37.7 | <0.0000001 | <0.1% |
| *hsa-miR-200b-3p* | 2251 | 34.6 | <0.0000001 | <0.1% |
| *hsa-miR-205-5p* | 509 | 31.7 | 0.0000646 | <0.1% |
| *hsa-miR-182-5p* | 2334 | 26.5 | <0.0000001 | <0.1% |
| *hsa-miR-142-3p* | 464 | 25.4 | 0.0000243 | <0.1% |
| *hsa-miR-148a-3p* | 470 | 19.8 | 0.0000417 | <0.1% |
| *hsa-miR-130b-3p* | 456 | 18.2 | 0.0000107 | <0.1% |
| *hsa-miR-424-5p* | 604 | 18 | 0.0000002 | <0.1% |
| *hsa-miR-19a-3p* | 395 | 17.8 | 0.0000818 | <0.1% |
| *hsa-miR-20a-5p* | 580 | 16.9 | 0.000002 | <0.1% |
| *hsa-miR-200c-3p* | 2300 | 16.1 | 0.0000061 | <0.1% |
| *hsa-miR-224-5p* | 2099 | 15 | 0.000005 | <0.1% |
| *hsa-miR-106b-5p* | 442 | 15 | 0.0002048 | <0.1% |
| *hsa-miR-34c-5p* | 428 | 14 | 0.0000129 | <0.1% |
| *hsa-miR-19b-3p* | 396 | 14 | 0.0001866 | <0.1% |
| *hsa-miR-512-3p* | 1823 | 13.9 | 0.0000037 | <0.1% |
| *hsa-miR-21-5p* | 397 | 13.1 | 0.0000144 | <0.1% |
| *hsa-miR-598-3p* | 1988 | 12.9 | 0.0000164 | <0.1% |
| *hsa-miR-362-3p* | 2117 | 11.9 | 0.000017 | <0.1% |
| *hsa-miR-642a-5p* | 1592 | 10.8 | 0.0000431 | <0.1% |
| *hsa-miR-517a-3p* | 2402 | 10.6 | 0.000042 | <0.1% |
| *hsa-miR-517c-3p* | 1153 | 10.3 | 0.0000153 | <0.1% |
| *hsa-miR-32-5p* | 2109 | 10.2 | 0.0001139 | <0.1% |
| *hsa-miR-340-5p* | 2258 | 10.2 | 0.0003355 | 0.001 |
| *hsa-miR-183-5p* | 2269 | 10.1 | 0.0000031 | <0.1% |
| *hsa-miR-337-5p* | 2156 | 10 | 0.0000458 | <0.1% |
| *hsa-miR-335-5p* | 546 | 9.7 | 0.000704 | 0.003 |
| *hsa-miR-18a-5p* | 2422 | 9.5 | 0.0007671 | 0.003 |
| *hsa-miR-138-5p* | 2284 | 9.3 | 0.0086037 | 0.024 |
| *hsa-miR-29b-3p* | 413 | 8.9 | 0.0047868 | 0.014 |
| *hsa-miR-301a-3p* | 528 | 8.8 | 0.0005242 | 0.002 |
| *hsa-miR-660-5p* | 1515 | 8.1 | 0.0027632 | 0.009 |
| *hsa-miR-708-5p* | 2341 | 8 | 0.0000013 | <0.1% |
| *hsa-miR-500a-5p* | 2428 | 7.9 | 0.0017319 | 0.006 |
| *hsa-miR-25-3p* | 403 | 7.9 | 0.0010505 | 0.004 |
| *hsa-miR-590-5p* | 1984 | 7.7 | 0.0079344 | 0.022 |
| *hsa-miR-452-5p* | 2329 | 7.2 | 0.0013239 | 0.005 |
| *hsa-miR-223-3p* | 2295 | 7.2 | 0.0004992 | 0.002 |
| *hsa-miR-130a-3p* | 454 | 7.1 | 0.0052755 | 0.015 |
| *hsa-miR-652-3p* | 2352 | 6.6 | 0.003254 | 0.01 |
| *hsa-miR-505-3p* | 2089 | 6.2 | 0.0002208 | <0.1% |
| *hsa-miR-185-5p* | 2271 | 5.8 | 0.0057977 | 0.016 |
| *hsa-miR-519a-3p* | 2415 | 5.8 | 0.000699 | 0.003 |
| *hsa-miR-17-5p* | 2308 | 5.4 | 0.000137 | <0.1% |
| *hsa-miR-362-5p* | 1273 | 5.1 | 0.002025 | 0.007 |
| *hsa-miR-221-3p* | 524 | 4.4 | 0.0000252 | <0.1% |
| *hsa-miR-106a-5p* | 2169 | 4.1 | 0.0000678 | <0.1% |
| *hsa-miR-502-5p* | 1109 | 3.9 | 0.0002395 | 0.001 |
| *hsa-miR-20b-5p* | 1014 | 3.8 | 0.000146 | <0.1% |
| *hsa-miR-181a-5p* | 480 | 3.7 | 0.0001548 | <0.1% |
| *hsa-miR-210-3p* | 512 | 3.5 | 0.0003412 | 0.001 |
| *hsa-miR-542-3p* | 1284 | 3.3 | 0.0099155 | 0.027 |
| *hsa-miR-146a-5p* | 468 | 3.2 | 0.0051031 | 0.015 |
| *hsa-miR-93-5p* | 1090 | 3.1 | 0.0000786 | <0.1% |
| *hsa-miR-34a-5p* | 426 | 3 | 0.0049853 | 0.015 |
| *hsa-miR-15b-5p* | 390 | 2.6 | 0.0002171 | <0.1% |
| *hsa-let-7b-5p* | 2619 | -2.1 | 0.0097662 | 0.027 |
| *hsa-miR-320a* | 2277 | -2.5 | 0.0027268 | 0.009 |
| *hsa-miR-574-3p* | 2349 | -3.2 | 0.0008842 | 0.003 |
| *hsa-miR-197-3p* | 497 | -3.8 | 0.0000391 | <0.1% |
| *hsa-miR-342-3p* | 2260 | -5.3 | 0.0015213 | 0.005 |
| *hsa-let-7c-5p* | 379 | -5.3 | 0.0005424 | 0.002 |
| *hsa-miR-139-5p* | 2289 | -5.9 | 0.0000303 | <0.1% |
| *hsa-miR-145-5p* | 2278 | -7.7 | 0.0001914 | <0.1% |
| *hsa-miR-133a-3p* | 2246 | -7.7 | 0.0053003 | 0.015 |
| *hsa-miR-125a-3p* | 2199 | -8.3 | 0.0009091 | 0.003 |
| *hsa-miR-139-3p* | 2313 | -11.8 | 0.0014051 | 0.005 |
| *hsa-miR-134-5p* | 1186 | -13.3 | 0.0000003 | <0.1% |
| *hsa-miR-486-5p* | 1278 | -34.5 | 0.0000318 | <0.1% |
| *hsa-miR-874-3p* | 2268 | -35.7 | 0.000448 | 0.002 |
| *hsa-miR-299-5p* | 600 | -38.5 | 0.0001542 | <0.1% |
| *hsa-miR-486-3p* | 2093 | -52.6 | 0.0001548 | <0.1% |
| *hsa-miR-891a-5p* | 2191 | -149.3 | 0.0000158 | <0.1% |

**Legend:** FC: Fold change; FDR: False discovery ratio; P value obtained from two-sample t-test.
