# Supplementary material for: Integrative miRNA and mRNA analysis in penile carcinomas reveals markers and pathways with potential clinical impact
Source: Oncotarget. 2017 Jan 21;8(9):15294–306. doi: 10.18632/oncotarget.14783 (PMC5362487; doi:10.18632/oncotarget.14783)
Supplement: Supplementary file 3 [file oncotarget-08-15294-s003.docx]

**Table S2.** The integrative analysis resulted in 598 interactions with negative correlation involving 68 miRNAs and 255 mRNA (miRWalk2.0 and/or miRTarBase). In bold, mRNA and miRNA experimentally validated (miRtarbase).

| ***miRNA*** | | | ***Target Gene*** | |
| --- | --- | --- | --- | --- |
| ***Down-expressed*** | | ***Overexpressed*** | | |
| ***hsa-miR-299-5p*** | ***SPP1*** | | | |
| ***hsa-miR-874-3p*** | ***AQP3*** | | | |
| ***hsa-miR-134-5p*** | ***FOXM1*** | | | |
| ***hsa-miR-145-5p*** | ***KLF5****,* ***CDK6****,* ***NRAS****,* ***SOCS7****,* ***HMGA2****,* ***MMP12****,* ***MMP1****, ONECUT2, SOX11, SLC38A2* | | | |
| *hsa-miR-139-5p* | *UHMK1* | | | |
| ***hsa-let-7c-5p*** | ***HMGA2****,* ***NRAS****, ONECUT2, FGD6, IGF2BP2, IGF2BP3* | | | |
| ***hsa-miR-197-3p*** | ***PMAIP1*** | | | |
| *hsa-miR-320a* | *ONECUT2, SOX11, IGF2BP3, SHCBP1* | | | |
| ***hsa-let-7b-5p*** | ***HMGA2****,* ***CDC25A****,* ***IGF2BP2****,* ***CDK6****,* ***CCNA2****,* ***HMGA1****, ONECUT2* | | | |
| ***Overexpressed*** | ***Down-expressed*** | | | |
| ***hsa-miR-31-5p*** | ***LATS2****,* ***TBXA2R****,* ***DMD****,* ***WASF3****,* ***DACT3****, AR, DNMT3A, MAP1B, CACNB2, SLC2A4* | | | |
| ***hsa-miR-135b-5p*** | ***LZTS1****, MAPRE2, FRMPD4, ARHGAP6* | | | |
| ***hsa-miR-141-3p*** | ***DLX5****,* ***TCF7L1****, OLFM1, ZCCHC24, TTR, LUC7L3, CUL3, TMEM110, TMEM170B, IRS2, KLF9, DLC1* | | | |
| ***hsa-miR-429*** | ***ZEB1****,* ***RERE****,* ***BCL2****,* ***TCF7L1****,* ***ZFPM2****, ATP2A2, NTF3, ZDHHC17, PSIP1, PRKCB, NAP1L5, PDS5B, ZC3H6, TMEM170B, JAZF1, KLF9, DLC1* | | | |
| ***hsa-miR-200a-3p*** | ***ZEB1****,* ***TCF7L1****,* ***SRF****,* ***DLX5****,* ***WASF3****,* ***ZFPM2****, OLFM1, TMEM110, ZCCHC24, IRS2, CUL3, SOX5, TTR, JAZF1, TMEM170B, LONRF2, FOXP1* | | | |
| ***hsa-miR-363-3p*** | ***S1PR1****, ATP2A2, ACTC1* | | | |
| *hsa-miR-455-5p* | *NR4A2, KDR, MYLIP, CRTAC1* | | | |
| ***hsa-miR-203a-3p*** | ***EYA4, LIFR*** | | | |
| *hsa-miR-455-3p* | *CUL3, SLC35F1* | | | |
| ***hsa-miR-200b-3p*** | ***ZEB1****,* ***CDKN1B****,* ***ZFPM2****,* ***ROCK2****,* ***RAB23****,* ***BTC****,* ***BCL2****,* ***RERE****,* ***TCF7L1****,* ***DLC1****, DUSP1, JAZF1, PPM1B* | | | |
| ***hsa-miR-205-5p*** | ***ZEB1****,* ***ZEB2****,* ***BCL2****,* ***PTPRM****,* ***LAMC1****,* ***LRRK2****,* ***CTGF****,* ***CYR61****,* ***AR*****,* ***BCL6****, CFL2, TRAK2, PLCB1, CADM1* | | | |
| ***hsa-miR-182-5p*** | ***MITF****,* ***RECK****,* ***SMARCD3****, NPTX1, CELF2, JAZF1, NRN1, TMOD1, RAB6B, FZD3, MEF2C* | | | |
| ***hsa-miR-142-3p*** | ***ABCG2****,* ***LRRC32****, CFL2* | | | |
| ***hsa-miR-148a-3p*** | ***ROCK1****,* ***S1PR1****, NPTX1, MEOX2, PRICKLE2, ZDHHC17, GPM6A* | | | |
| ***hsa-miR-130b-3p*** | ***PPARGC1A****,* ***PDGFRA****,* ***ZBTB4****,* ***CSF1****,* ***PPARG*****, CCDC85A, MEOX2, S1PR1, NOL4, ADCY1, RAB9B* | | | |
| ***hsa-miR-424-5p*** | ***FGFR1****,* ***FGF2****,* ***NFIA****, SKI, SH3BGRL2, RELN, LRP1B, MYLK, PKDCC, TGFBR3, LAMC1* | | | |
| ***hsa-miR-19a-3p*** | ***MECP2****,* ***MYCN****,* ***KIT****,* ***NR4A2****,* ***ZBTB4****,* ***ESR1****,* ***ERBB4****, CUL3, ZMYND11, TSHZ3, TGFBR3, PRICKLE2, MYLIP, UBL3, LONRF1* | | | |
| ***hsa-miR-20a-5p**** | ***PRKG1****,* ***PPARG*****,* ***MAP3K12****,* ***FBXO31****,* ***TCEAL1****,* ***RGS5****,* ***EPAS1****,* ***KIT****,* ***BCL2****, NTN4, HECTD2, ZFPM2, MYCN, TAL1, RASL11B, YPEL2, PPP3CA, CRY2, CFL2, TCF7L1, ATP1A2, PKD2, FZD3, TXNIP, RRAGD, EZH1, ZBTB4* | | | |
| ***hsa-miR-200c-3p*** | ***ZEB1****,* ***ZEB2****,* ***FBLN5****,* ***ROCK2****,* ***NTRK2****,* ***NTF3****,* ***CFL2****,* ***TCF7L1****,* ***KLF9****,* ***BCL2****,* ***BTC****,* ***EDNRA****,* ***DLC1****, ATP2A2, CYP1B1, NAP1L5, ZC3H6, PSIP1, PDS5B, TMEM170B, DUSP1, JAZF1, WASF3, NOG* | | | |
| *hsa-miR-203a-3p* | *COL21A1, NUAK1, SEC62, LAMC1, GUCY1A3, LRCH2* | | | |
| ***hsa-miR-224-5p**** | ***TCEAL1****, TMEM110, CASC3* | | | |
| ***hsa-miR-106b-5p**** | ***TCEAL1****,* ***ZBTB4****, MYCN, YPEL2, GUCY1A3, RASL11B, NTN4, NR4A3, ATP1A2, CALD1, EPAS1, TSHZ3, TXNIP, PDGFRA, NR4A2, FZD3* | | | |
| ***hsa-miR-19b-3p*** | ***MYCN****,* ***MYLIP****,* ***ESR1****, ZNF521, SULF1, CUL3, TSHZ3, PRICKLE2, ZMYND11, TGFBR3, ZBTB4* | | | |
| ***hsa-miR-34c-5p*** | ***MYCN****,* ***NOTCH4****,* ***ITPR1****, NRN1, CELF2, FOXP1* | | | |
| *hsa-miR-512-3p* | *TOX, ADAMTS5, SMAD9, SPOP, FGL2, ZNF577* | | | |
| ***miRNA*** | | ***Target Gene*** | |  |
| ***Overexpressed*** | | ***Down-expressed*** | |  |
| ***hsa-miR-21-5p*** | | ***RECK****,* ***RHOB****,* ***LRRFIP1****,* ***NFIB****,* ***NTF3****,* ***NFIA****,* ***SOD3****,* ***FMOD****,* ***BCL6****,* ***SOX5****,* ***CLU****,* ***MEF2C****,* ***TGFBR3****, SKI* | |  |
| *hsa-miR-362-3p* | | *ZFHX4* | |  |
| *hsa-miR-642a-5p* | | *APPBP2* | |  |
| ***hsa-miR-340-5p*** | | ***MECP2****,* ***ROCK1****, PPM1B, LRP1B, KIAA2022, DMD, ADAMTS5* | |  |
| ***hsa-miR-32-5p*** | | ***REV3L****, ARHGEF17, ATP2A2, PDS5B, CELF2* | |  |
| ***hsa-miR-183-5p*** | | ***EGR1****,* ***DKK3****,* ***ZEB1****,* ***AKAP12****, HECTD2, CELF2, MEF2C* | |  |
| ***hsa-miR-335-5p*** | | ***FMN2****,* ***ROCK1****,* ***SOX17****,* ***DAAM2****, PRKAA2, PRDM2* | |  |
| ***hsa-miR-18a-5p*** | | ***ESR1****,* ***CTGF****,* ***BCL2****,* ***NEDD9****, TSHZ3, PHF2, TMEM170B* | |  |
| ***hsa-miR-138-5p*** | | ***RELN****, ZMYND11, NPTX1, PCSK2, MYO5C, SLC35F1, FIGF* | |  |
| ***hsa-miR-29b-3p*** | | ***DNMT3A****,* ***PDGFRB****,* ***MYCN****,* ***TGFB3****,* ***BACE1****,* ***LOXL4****,* ***ESR1****,* ***PDGFRA****, COL21A1, KIAA2022, PKNOX2, LAMC1, MEX3B* | |  |
| ***hsa-miR-301a-3p*** | | ***MEOX2****, NOL4, S1PR1, CCDC85A, PLCB1, ZFPM2, SULF1, PDGFRA, PPARG*,* | |  |
| *hsa-miR-660-5p* | | *EPAS1, RFX3, PCSK2, KLF9* | |  |
| ***hsa-miR-708-5p*** | | ***ZEB2****,* ***BCL2****, AMIGO1* | |  |
| ***hsa-miR-25-3p*** | | ***TCEAL1****,* ***ATP2A2****,* ***LATS2****,* ***REV3L****,* ***RECK****,* ***CDKN1C****, TECPR2, NPTX1* | |  |
| *hsa-miR-500a-5p* | | *LUC7L3, RFX3, TMEM170B, CELF2* | |  |
| *hsa-miR-590-5p* | | *SKI, CDH6, CEP68* | |  |
| ***hsa-miR-452-5p*** | | ***CDKN1B****, PDZRN3, EPS8, FAM129A, IRS2, PPM1B, XYLT1* | |  |
| ***hsa-miR-223-3p*** | | ***NFIA****,* ***FOXO1****,* ***LPIN2****,* ***NFIX****,* ***SLC2A4****,* ***TAL1****,* ***TOX****,* ***MEF2C****, SCN3A, MAP1B, ZFHX3* | |  |
| ***hsa-miR-130a-3p*** | | ***MEOX2****,* ***ESR1****, CCDC85A, S1PR1, PLCB1, ADCY1, RAB9B, PDGFRA, LAMC1* | |  |
| *hsa-miR-505-3p* | | *VGLL3* | |  |
| *hsa-miR-519a-3p* | | *MAP1B, ZBTB4, SMOC2, PPP3CA, AHCTF1, RFX3, RASL11B, PKD2, PDGFRA* | |  |
| ***hsa-miR-185-5p*** | | ***SIX1****,* ***EPAS1****,* ***AR*****, SOX13, SPTBN1, PDS5B, CACNB1, ATP2A2, CELF2, DTNA* | |  |
| ***hsa-miR-17-5p*** | | ***BCL2****,* ***TCEAL1****,* ***FBXO31****,* ***CLU****,* ***HSPB2****,* ***MAP3K12****,* ***PKD2****,* ***ZBTB4****,* ***EPAS1****, NBEA, YPEL2, ATP1A2, NTN4, SLC16A12, SMOC2, MYCN, NOL4, NR4A3, EZH1, ZFPM2, PPP3CA, TXNIP, TAL1, CFL2, TSHZ3, NR4A2, JAZF1, FZD3* | |  |
| *hsa-miR-362-5p* | | *LUC7L3, DMD, TMEM47* | |  |
| ***hsa-miR-221-3p*** | | ***CDKN1C****,* ***TCEAL1****,* ***HECTD2****,* ***RECK****,* ***PIK3R1****,* ***MEOX2****,* ***ESR1****,* ***ZEB2****, NAP1L5, PKDCC, KDR, PHF2, ZFPM2* | |  |
| ***hsa-miR-106a-5p*** | | ***MYLIP****,* ***TGFBR2****, NBEA, TNRC6C, NTN4, ATP1A2, CALD1, YPEL2, SLC16A12, SALL1, EZH1, NR4A3, MYCN, PPP3CA, CFL2, NOL4, TAL1, TSHZ3, PKD2, ZBTB4, FGL2, EPAS1, FZD3, PDGFRA* | |  |
| *hsa-miR-502-5p* | | *PRDM2* | |  |
| ***hsa-miR-20b-5p*** | | ***PPARG*****,* ***MYLIP****,* ***ESR1****, NBEA, YPEL2, ATP1A2, NTN4, SMOC2, NR4A3, MYCN, NOL4, PPP3CA, TXNIP, SLC16A12, PKD2, EPAS1, TSHZ3, FGL2, PDGFRA, RASL11B, CFL2, TAL1, ZBTB4, JAZF1, NR4A2* | |  |
| ***hsa-miR-181a-5p*** | | ***ABCG2****,* ***WIF1****,* ***PROX1****,* ***PPP3CA****,* ***RGS5****, GLS, SOX5, ATP2A2, CUL3, CBX7, CACNB2, KIAA2022, ZFP36L2, AHCTF1* | |  |
| ***hsa-miR-210-3p*** | | ***DDAH1****,* ***FOXN3****,* ***EHD2****,* ***HIF3A****,* ***SH3BGRL****,* ***INPP5A****,* ***GPD1L****, CUL3* | |  |
| *hsa-miR-542-3p* | | *MAP1B* | |  |
| ***hsa-miR-146a-5p*** | | ***ERBB4****,* ***ROCK1****,* ***DUSP1****, FZD3* | |  |
| ***hsa-miR-93-5p*** | | ***SLC2A4****,* ***LATS2****,* ***ZBTB4****,* ***TGFBR2****, YPEL2, NTN4, NBEA, ATP1A2, SMOC2, RASL11B, MYCN, NR4A3, CFL2, HECTD2, PPP3CA, EZH1, SLC16A12, EPAS1, TAL1, TXNIP, NR4A2, TSHZ3, JAZF1* | |  |
| ***hsa-miR-34a-5p*** | | ***MYCN****,* ***PDGFRB****,* ***AXL****,* ***AR*****,* ***KIT****, PLEKHH2, SCN2B, ZFHX4, XYLT1, CAPN6* | |  |
| ***hsa-miR-15b-5p*** | | ***RECK****,* ***KDR****, MYLK, ZFHX4, CUL3, MEOX2, ADAMTS5, RAB9B, PDK4, TGFBR3, SH3BGRL2, EYA1, ITPR1* | |  |

The integrative analysis based only in experimentally confirmed miRNA-mRNA interactions (miRTarbase) revealed 53 miRNAs and 130 potential mRNA targets (222 interactions).
